# ﻿*Violasuavis* var. *﻿pannonica* (Violaceae), a new white-flowered violet from central Europe

**DOI:** 10.3897/phytokeys.242.121734

**Published:** 2024-05-09

**Authors:** Iva Hodálová, Pavol Mereďa Jr

**Affiliations:** 1 Institute of Botany, Plant Science and Biodiversity Centre, Slovak Academy of Sciences, Dúbravská cesta 9, SK-845 23 Bratislava, Slovakia Institute of Botany, Plant Science and Biodiversity Centre, Slovak Academy of Sciences Bratislava Slovakia

**Keywords:** Pannonia, protologue, taxonomy, variety

## Abstract

Violasuavisvar.pannonica (Violaceae) from central Europe is described here as a new variety to science. It is most similar to the blue-flowered V.suavisvar.suavis and the white-flowered V.suavisvar.catalonica and V.suavissubsp.naqshii, but exhibits differences in several characters, such as petal colour, spur shape, fimbriae length on the stipules, bracteoles position on the peduncle and lamina sinus shape. Although the new taxon is often considered a colour mutation of V.suavisvar.suavis, previous genetic analyses revealed that these white-flowered plants do not arise recurrently at different locations (having multiple origins), but rather form a monophyletic evolutionary lineage. To date, the occurrence of V.suavisvar.pannonica has been reported in the Slovak Republic, the Czech Republic and western Ukraine. In this paper, we report its occurrence in Austria and Hungary. Notes on its etymology, distribution, ecology, origin and hybridization, as well as photographs of the new variety (including the holotype) are also provided.

## ﻿Introduction

*Violasuavis* M.Bieb. from ViolaL.subsect.Viola (Violaceae) is a perennial herb, differing from related Viola species of the subsection in a number of characters: relatively short and stout stolons, long fimbriate stipules, bracteoles located below the middle of the peduncle, glabrous calycine appendages appressed to the peduncle, fragrant flowers (cf. Becker 1910; Gams 1925; Valentine et al. 1968; Kirschner and Skalický 1990; Marcussen and Nordal 1998; Hodálová et al. 2008; Mereďa et al. 2008a, 2008b), and 2n = 40 chromosomes (interpreted by different authors either as tetraploid- or (paleo)octoploid; cf. Schmidt 1961; Kirschner and Skalický 1990; Mereďa et al. 2008a, 2008b; Marcussen et al. 2022). The species has been described from northeastern Ukraine (town of Merefa near the city of Kharkiv; the type is deposited in the herbarium of the Komarov Botanical Institute of The Russian Academy of Sciences, Saint Petersburg, Russia; herbarium acronym LE; Marschall von Bieberstein 1819). The distribution range of *V.suavis* extends from the Caucasus and Ural Mountains through the European Mediterranean and sub-Mediterranean regions to the Iberian Peninsula and Morocco; secondarily, due to cultivation, its area also extends to some parts of western, central and northern Europe (Marcussen and Nordal 1998; Mereďa et al. 2011). An isolated occurrence of the species has been recently reported in Kashmir Himalaya (Ganie et al. 2023). *Violasuavis* prefers dry grasslands, shrublands and open deciduous forests; in addition, it frequently occurs in human-made or human-influenced habitats, such as gardens, parks and cemeteries (Kirschner and Skalický 1990; Marcussen and Nordal 1998; Hodálová et al. 2008; Mereďa et al. 2008b).

*Violasuavis* is a polymorphic species displaying geographically correlated morphological variation, which complicates its taxonomic treatment (Haesler 1975; Marcussen and Nordal 1998; Marcussen and Borgen 2000; Hodálová et al. 2008; van den Hof et al. 2008; Mereďa et al. 2008a, 2011; Ganie et al. 2023). The results of our previous molecular, morphological and chorological studies (Hodálová et al. 2008; Mereďa et al. 2008a, 2008b, 2011) provided strong support for the recognition of four major genetic-morphological lineages of *V.suavis* in Europe, three of which are currently treated in the majority of relevant studies at the subspecific level (e.g., Raab-Straube and Henning 2018+; Nikolić 2020; however, see Marcussen et al. 2022, according to which taxa within *V.suavis* do not merit formal taxonomic recognition): (1) V.suavissubsp.suavis, occurring in central and eastern Europe (e.g., Mereďa et al. 2008b; Danihelka 2019; Pyšek et al. 2022); (2) V.suavissubsp.adriatica (Freyn) Haesler (Haesler 1975: 111; bas. *V.adriatica*Freyn 1884: 679), occurring in northeastern Italy, southwestern Slovenia and northwestern Croatia (Mereďa et al. 2011; Nikolić 2020); and (3) V.suavissubsp.austrodalmatica Mereďa & Hodálová, occurring in southern Croatia, southern Bosnia and Herzegovina and southwestern Montenegro (Mereďa et al. 2011; Nikolić 2020). All three of these subspecies are morphologically clearly distinguishable by several characters, mainly the leaf indument (Mereďa et al. 2008a, 2011).

The detailed morphological pattern of the fourth European genetic lineage (provisionally named *V.suavis* 'Spain') has not yet been elucidated and requires a more thorough study (see Mereďa et al. 2008a, 2011). Populations of this lineage have been found in northeastern Spain and potentially in the adjacent part of France. In the relevant literature, the taxon is regarded as identical to *V.suavis* s. str. (e.g., Valentine et al. 1968; Muñoz Garmendia et al. 1993) or V.suavissubsp.suavis (e.g., Raab-Straube and Henning 2018+), depending on the taxonomic species concept.

**Figure 1.**
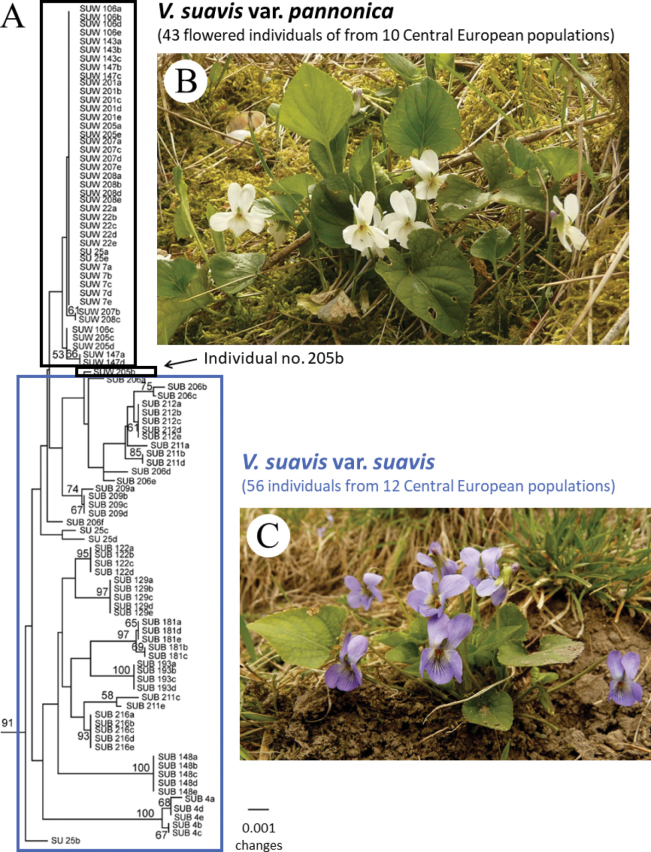
**A** Part of the neighbor-joining tree of AFLP data of 100 central European individuals of Violasuavissubsp.suavis using Nei and Li distance. Numbers above branches indicate bootstrap support above 50%. Accession labels include taxon abbreviation (SUB – blue-flowered variant (V.suavisvar.suavis); SUW – white-flowered variant (V.suavisvar.pannonica)) and population numbers. Taken from Mereďa et al. (2008a)**B**V.suavisvar.pannonica (Slovakia, Zbrojníky village; photographed by P. Mereďa Jr., 17 April 2013) **C**Violasuavisvar.suavis (Hungary, Visegrád village; photographed by P. Mereďa Jr., 26 March 2011).

While V.suavissubsp.adriatica and V.suavissubsp.austrodalmatica possess only blue to bluish violet petals, V.suavissubsp.suavis and *V.suavis* 'Spain' have two colour variants: one with typical blue to bluish violet petals (blue variant; Fig. 1C) and one with white petals (white variant; Fig. 1B). The blue and white variants within the given taxa are genetically distinct (Fig. 1A), and in addition to flower pigmentation, they differ in several other morphological characters (Mereďa et al. 2008a, 2008b, 2011; see also Diagnosis). Amplified fragment length polymorphism (AFLP) analyses revealed that white-flowered V.suavissubsp.suavis and white-flowered *V.suavis* 'Spain' do not arise recurrently at different locations (with multiple origins) but rather form two monophyletic, evolutionarily independent, parallel genetic entities descended from different blue-flowered progenitors in two distinct areas (central Europe and the Iberian Peninsula). Thus, the sympatric occurrence of two colour variants within both V.suavissubsp.suavis and *V.suavis* 'Spain' is most likely the result of secondary contact (Mereďa et al. 2008a).

**Figure 2.**
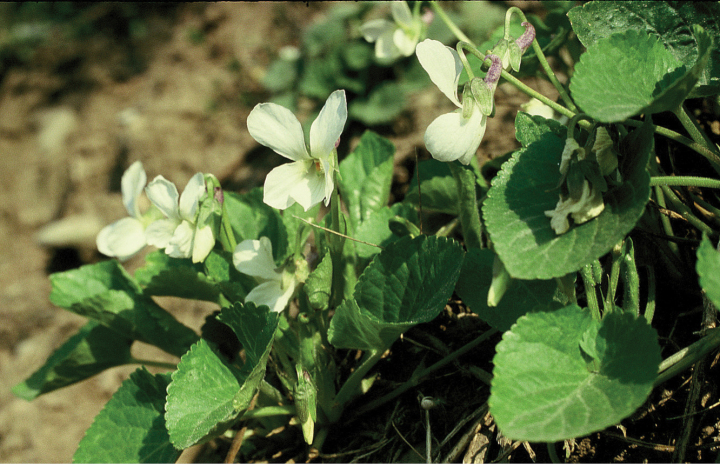
Violasuavisvar.catalonica (Spain, town of Manlleu; photographed by P. Mereďa Jr., 17 March 2006).

The white-flowered variant of *V.suavis* 'Spain' from the Iberian Peninsula (Fig. 2) was originally described as *V.catalonica* W.Becker (Becker 1929: 43) from the current public park Jardin del Turó del Putget in Barcelona (Spain) (the type is deposited in the Conservatoire et Jardin Botaniques de la Ville de Genève, Switzerland; herbarium acronym G); later, it was treated at the subspecific level [V.suavissubsp.catalonica (W.Becker) O.Bolòs & Vigo; Bolòs and Vigo 1974: 80] or as the variety V.suavisvar.catalonica (W.Becker) Espeut (Espeut 1999: 16).

The central European white-flowered variant of V.suavissubsp.suavis (Fig. 1B) has not yet been formally described. In local databases, floras and keys, it is neglected (Farkas 2009), treated as “white-flowered violets of presumed hybrid origin” (Kirschner and Skalický 1990; Suda 2002) or a “white-flowered morphotype” of V.suavissubsp.suavis (Mereďa et al. 2008b), or considered a *V.suavis* variation (e.g., Michalcová 2011+; Danihelka 2019; FloraVeg.EU 2022+).

**Figure 3.**
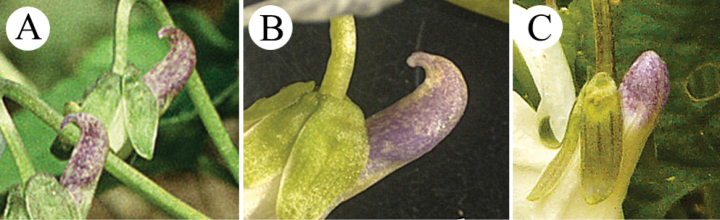
Shape of the spur **A**Violasuavisvar.catalonica (Spain, town of Manlleu; photographed by P. Mereďa Jr., 17 March 2006) **B**V.suavissubsp.naqshii (India, town of Hazratbal; taken from Ganie et al. 2023) **C**V.suavisvar.pannonica (Slovakia, Zbrojníky village; photographed by P. Mereďa Jr., 17 April 2013).

Recently, white-flowered individuals of *V.suavis* have been found in a third geographic area, the Kashmir Himalaya (India), and described as a new subspecies, V.suavissubsp.naqshii (Ganie et al. 2023). This taxon has not been studied morphometrically or genetically. However, it shares several common characters with V.suavisvar.catalonica, such as a hooked spur (Fig. 3A, B) and bracteoles located on the peduncle at a relatively high position.

The aim of this study was to formally describe the central European white-flowered populations of *V.suavis* as a separate taxon at the variety level, based on the results of our previous genetic and morphological studies of the genus *Viola* (Mereďa et al. 2008a, 2011).

## ﻿Material and methods

**Figure 4.**
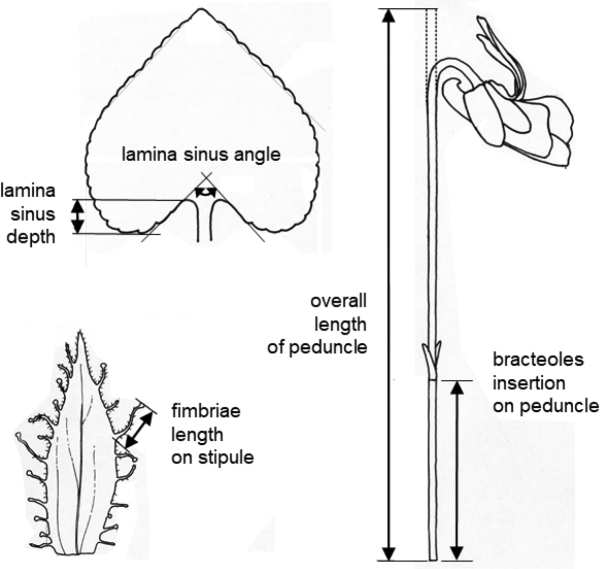
Selected morphological characters used in Diagnosis (after Hodálová et al. 2008).

Living plant material was used for morphological studies, including 173 individuals from 16 populations of Violasuavisvar.suavis, 108 individuals from 12 populations of V.suavisvar.pannonica and 42 individuals from 5 populations of V.suavisvar.catalonica. Whenever possible, three measurements were made for each vegetative character, and two measurements were made for each floral character. The value ranges represent the 10^th^ and 90^th^ percentiles, with the 1^st^ and 99^th^ percentiles in parentheses. All measurements were performed at the time of flowering; for character explanations, see Fig. 4. Details on the origin of the material used are given in Mereďa et al. (2008a). Character values for V.suavissubsp.naqshii have been taken from Ganie et al. (2023).

## ﻿Taxonomy

### ﻿ Viola suavis var. pannonica

Taxon classificationPlantaeMalpighialesViolaceae

Hodálová & Mereďa var. nov.

04061C79-8387-5931-B5C9-377C81F1F52F

urn:lsid:ipni.org:names:77341546-1

**Figure 5.**
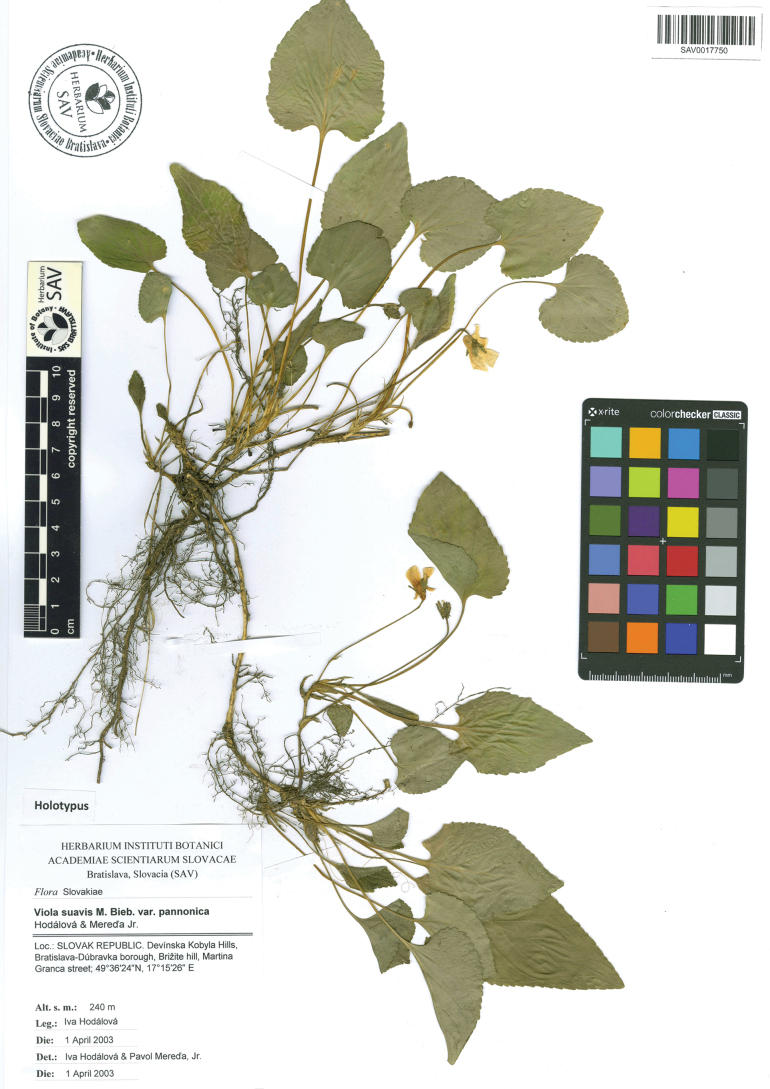
The holotype specimen of Violasuavisvar.pannonica deposited in the herbarium of the Institute of Botany, Slovak Academy of Sciences (herbarium acronym SAV).

**Figure 6.**
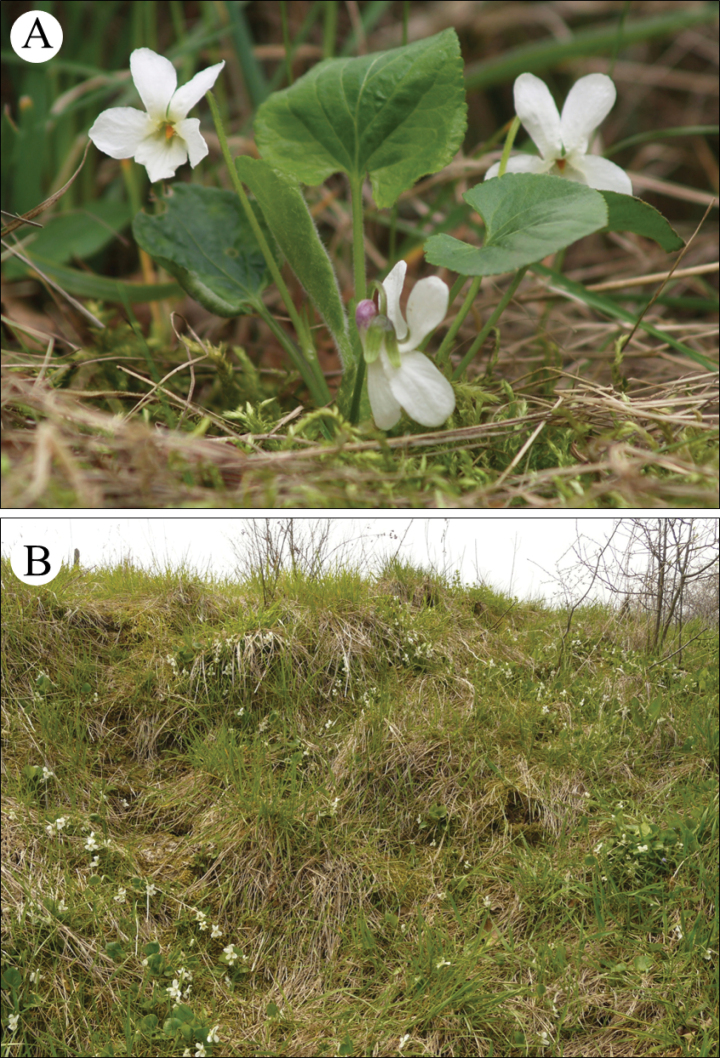
**A**Violasuavisvar.pannonica, plant from the type population (Slovak Republic, Bratislava-Dúbravka borough; photographed by P. Mereďa Jr., 3 April 2008) **B**V.suavisvar.pannonica, habitat (Slovak Republic, Zbrojníky village; photographed by P. Mereďa Jr., 17 April 2013).

Figs 1B , 3C , 5 , 6

#### Diagnosis.

Violasuavisvar.pannonica can be unambiguously distinguished from V.suavisvar.suavis by flower colour: V.suavisvar.pannonica possesses white petals (and a pale to deep violet spur, very rarely whitish or slightly yellow-greenish; Figs 1B, 3C, 6A); V.suavisvar.suavis possesses pale to deep blue or bluish violet petals with a large conspicuous white throat at the base, covering 1/3–1/2 of the length of the lateral and anterior petals (and a pale to deep violet spur; Fig. 1C). In addition to characters associated with pigmentation of the generative and vegetative parts, both varieties also differed in terms of the length of the fimbriae on the stipules ((0.8–)1.2–2.5(–3.1) mm long in V.suavisvar.pannonica vs. (0.4–)0.7–2.0(–2.8) mm long in V.suavisvar.suavis) and by the position of the bracteoles on the peduncle (at (4–)9–29(–40)% of the overall length of the peduncle in V.suavisvar.pannonica vs. (7–)13–42(–52)% in V.suavisvar.suavis).

In addition, V.suavisvar.pannonica differs from the white-flowered V.suavisvar.catalonica and V.suavissubsp.naqshii, having straight or only slightly upward-curved spur at the top (Fig. 3C) (vs. often distinctly hooked spur (curved up or backwards at the top) in V.suavisvar.catalonica and V.suavissubsp.naqshii; Fig. 3A, B). Moreover, V.suavisvar.pannonica differs from V.suavisvar.catalonica in terms of the lamina sinus angle ((0–)35–105(–135)° in V.suavisvar.pannonica vs. (-90–)-23–45(–85)° in V.suavisvar.catalonica), lamina sinus depth ((0.1–)0.2–0.55(–1.4) cm vs. (0.3–)0.45–1.1(–1.4) cm), and bracteoles located near the base of the peduncle (at (4–)9–29(–40)% of the overall length of the peduncle vs. at (15–)25–52(–60)% of the overall length of the peduncle). Violasuavisvar.pannonica differs from V.suavissubsp.naqshii in its longer petiole hairs (0.2–0.6(–0.8) mm in V.suavisvar.pannonica vs. up to 0.1 mm in V.suavissubsp.naqshii).

#### Type.

Slovak Republic. Devínska Kobyla Hills, Bratislava-Dúbravka borough, Brižite hill, Martina Granca street, 48°11′49″N, 17°01′29″E, elev. 240 m, 1 April 2003, I. Hodálová (Holotype: SAV (SAV0017750; Fig. 5)).

#### Phenology.

Flowering from March to April.

#### Etymology.

The epithet “pannonica” refers to the geographical region of Pannonia, which is the centre of the hitherto known distribution of the new variety (namely southeastern Moravia in the Czech Republic, northeastern Austria, southern Slovakia, the southern part of Zakarpattia Oblast in western Ukraine, and Hungary; see Distribution).

#### Distribution.

**Figure 7.**
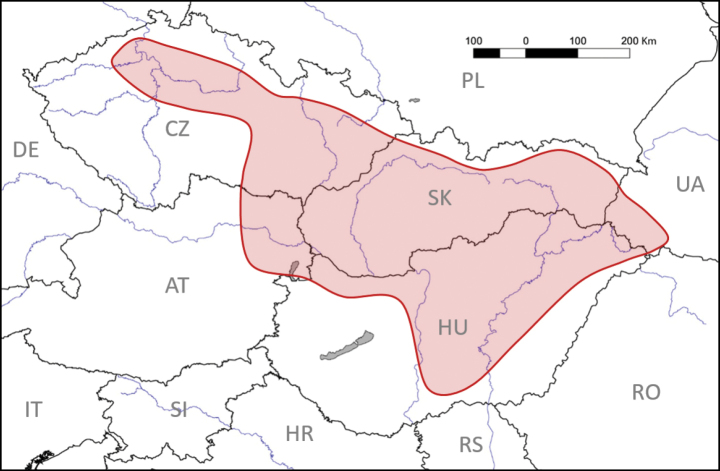
Distribution of Violasuavisvar.pannonica.

This variety has been reported in the Slovak Republic (Hodálová et al. 2008; Mereďa et al. 2008a; detailed distribution is given in Mereďa et al. 2008b; see also photographs of *V.suavis* at iNaturalist.org (Photo ID: 202520428, 201130424), nahuby.sk (Photo ID: 257298), http://flora.upol.cz/fotogalerie/info/9125-Viola-suavis/0-42.html, and www.botany.cz/cs/viola-suavis), the Czech Republic (Kirschner and Skalický 1990: 402; Suda 2002: 214 – both papers report the taxon in the note for *V.alba* as “white-flowered violets of presumed hybrid origin”; Hodálová et al. 2008; Mereďa et al. 2008a; Michalcová 2011+; Danihelka 2019; FloraVeg.EU 2022+; see also photographs of *V.suavis* at iNaturalist.org (Photo ID: 151489049, 151956929, 189937608, 201837659, 201843269, 201843461, 201905803, 201906388, 202177193, 202414626, 202716834, 202963367)), western Ukraine (Mereďa et al. 2008a), Austria, and Hungary (both this paper; Fig. 7).

This new variety has not yet been reported in the territories of Austria (cf. Fischer et al. 2008) and Hungary (cf. Farkas 2009). Its occurrence in Austria is documented by the photograph from Martin A. Prinz from the town of Traiskirchen, available from iNaturalist.org (Photo ID: 150078107, ut *V.suavis*). In Hungary we found V.suavisvar.pannonica in one location in the northern part of the country: Visegrád village, at the end of Kálvária street, 47°47′09″N, 18°58′20″E, 126 m, 26 March 2011, P. Mereďa Jr. (photo). In addition, we examined seven other herbarium specimens of the taxon from Hungary (all deposited in the Hungarian Natural History Museum, Budapest, Hungary; herbarium acronym BP; localities are arranged from west to east): near the town of Győr, Csanak, 16 April 1917 and 21 April 1928, S. Polgár (BP), both ut *Violaalba*; Piliscsaba village, Disznófő hill, 6 April 1913, L. Vajda (BP), ut *V.alba*; Budapest city, district II, Hármashatárhegy hill, 12 March 2000, É. I. Bőhm (BP), ut *V.odorata*; Budapest city, district II, Szemlőhegy utca street, 2 April 1917, Á. Boros (BP), ut *V.odorata*; Sárospatak town, 10 April 1933, Á. Kiss (BP), ut *V.alba*; and near the town of Nyiregyháza, Nyiregyházai-erdő forest, 25 March 1927, Á. Boros (BP), ut *V.suavis*. In Hungary this variety has also been documented in two photographs available from iNaturalist.org (Photo ID: 201304283, 203637437). The precise locations of the new variety in the Czech Republic, Austria, Ukraine and Hungary are not yet known, and in these countries, this violet will certainly be more widespread than the data published thus far indicate. The occurrence of V.suavisvar.pannonica is also expected in other central European countries.

#### Ecology.

It grows (often in extensive patches; Fig. 6B) mainly in human-made or human-influenced habitats, such as lawns, parks, cemeteries and roadsides in human settlements. It is also commonly cultivated in gardens, from which it is escaping (due to efficient spreading by rooting procumbent stolons and a high seed set per capsule) into their vicinity. In contrast to the nominate variety, V.suavisvar.pannonica only rarely extends into natural and semi-natural habitats outside settlements, where it grows in dry to mesophilous grasslands (Fig. 6B), shrublands, forest edges and open deciduous forests on various soil types (Mereďa et al. 2008a, 2008b).

#### Taxonomic notes and hybridisation.

Although in some older studies the authors assumed a hybrid origin of V.suavisvar.pannonica (based mainly on some shared morphological characters with other members of V.subsect.Viola, namely, *V.odorata* L., *V.collina* Besser, *V.hirta* L., *V.suavis*, and *V.alba* Besser; cf. Kirschner and Skalický 1990; Suda 2002), molecular analyses clearly revealed that the variety is exclusively derived from the typical blue-flowered central European V.suavissubsp.suavis (similar to V.suavisvar.catalonica, which is derived from the Iberian blue-flowered *V.suavis* 'Spain'; Mereďa et al. 2008a, 2011). Genetic analyses revealed that V.suavisvar.pannonica has very low genetic variation (Fig. 1A), and most populations in central Europe likely represent the same strain, which was spread in the past by cultivation as ornamentals in gardens and parks and subsequently escaped into the surrounding natural environment. The highly reduced genetic diversity and absence of unique AFLP fragments in individuals of V.suavisvar.pannonica, along with the patterns of fragment sharing and population clustering, clearly demonstrate that the origin of V.suavisvar.pannonica has been recent, perhaps within the last few centuries (Mereďa et al. 2008a).

Although V.suavisvar.pannonica is often sympatric with the blue-flowered V.suavisvar.suavis as well as other species of Violasubsect.Viola (especially *V.odorata* and *V.hirta*), no morphologically or genetically intermediate individuals have been detected. The absence of hybrids with *V.odorata* and *V.hirta* is not surprising as both these species have a different number of chromosomes (2n = 20), and heteroploid hybrids are rare in Violasubsect.Viola (Mereďa et al. 2008b).

However, the absence of morphologically or genetically intermediate plants between V.suavisvar.suavis and V.suavisvar.pannonica is especially surprising. It is possible that the flower colour in violets may be encoded by a biallelic system where the blue allele is dominant; in that case, colour intermediates might not be possible. However, it is surprising that even in the AFLP analyses, we practically did not identify genetically intermediate individuals, even in locations where the blue- and white-flowered plants were found growing in close proximity or even partly intermingled (population nos 25 and 27, 205 and 206, 208 and 209; Mereďa et al. 2008a and Fig. 1A). An identical situation also occurred for the two colour variants of *V.suavis* in Spain (Mereďa et al. 2008a). The AFLP analyses indicated a possible hybrid origin in only one plant, white-flowered individual no. 205b, which grouped together (although with low bootstrap support) with the blue-flowered plants (Mereďa et al. 2008a and Fig. 1A).

## Supplementary Material

XML Treatment for Viola suavis var. pannonica

## ﻿Additional information

### Author ORCIDs

Iva Hodálová https://orcid.org/0000-0002-9905-381X

Pavol Mereďa Jr https://orcid.org/0000-0003-3702-7015

### Data availability

All of the data that support the findings of this study are available in the main text.
